# Knowledge, Perceptions and Behaviors Related to COVID-19 in a University Setting in Malaysia

**DOI:** 10.3389/fpubh.2022.873022

**Published:** 2022-04-11

**Authors:** Kai Wei Lee, Sook Fan Yap, Hooi Tin Ong, Pooi Pooi Leong, Nadia Mohamad Hatta, Munn Sann Lye

**Affiliations:** ^1^Department of Pre-clinical Sciences, Faculty of Medicine and Health Sciences, Universiti Tunku Abdul Rahman, Kajang, Malaysia; ^2^Centre for Research on Communicable Diseases, Universiti Tunku Abdul Rahman, Kajang, Malaysia; ^3^Department of Population Medicine, Faculty of Medicine and Health Sciences, Universiti Tunku Abdul Rahman, Kajang, Malaysia

**Keywords:** COVID-19, knowledge, perception, self-risk, self-efficacy, behavior

## Abstract

**Background:**

In Malaysia the COVID-19 disease (COVID-19) has continued to escalate since its first detection in late January 2020, despite widespread implementation of control measures. This study aims to determine the knowledge, perception and behaviors with respect to COVID-19 in the midst of the third wave of the infection.

**Methods:**

A cross-sectional study was carried out among staffs and students of Universiti Tunku Abdul Rahman (UTAR). The survey consists of basic sociodemographic information, 22 items on knowledge on COVID-19, 3 items on perceived self-risk, 2 items on preparedness & perceived self-efficacy, 10 items on preventive (own) measures, 9 items assessing unwanted and desirable behaviors during the pandemic. Simple and multiple linear regression were performed to determine the factors associated with knowledge, preventive measures adopted, self-risk perception, preparedness & perceived self-efficacy, and behaviors.

**Results:**

A total of 434 responded to the survey of whom the majority (85.1%) had high scores for knowledge (mean score of 18.72 out of 22). A significant positive association was found between knowledge and older age (adjusted B coefficient (SE) = 0.046 (0.022), *p* = 0.039), those from medical faculty (adjusted B coefficient (SE) = 0.870 (0.420), *p* = 0.039) and residence in high-risk areas (adjusted B coefficient (SE) = 0.831 (0.295), *p* = 0.005). Predictors for higher perception of COVID-19 risk included presence of COVID-19 cases among social contacts (adjusted B coefficient (SE) = 0.751 (0.308), *p* = 0.015) and living with elderly (adjusted B coefficient (SE) = 1.137 (0.296), *p* < 0.001), while that for perception of preparedness and self-efficacy were living with children (adjusted Beta coefficient (SE) = 0.440 (0.173), *p* = 0.011) and absence of positive cases among social contacts (adjusted B coefficient (SE) = 0.418 (0.183), *p* = 0.023). Good preventive measures among the respondents were positively associated with knowledge (adjusted B coefficient (SE) = 0.116 (0.025), *p* < 0.001), as well as with female gender (adjusted B coefficient (SE) = 0.348 (0.142), *p* = 0.014). Unwanted behavior was significantly associated with male gender (adjusted B coefficient (SE) = 0.664 (0.321), *p* = 0.039) and COVID-19 positive status (adjusted B coefficient (SE) = 9.736 (3.297), *p* = 0.003). Knowledge of COVID-19 (adjusted B coefficient (SE) = 0.069 (0.035), *p* = 0.048) and being married (adjusted B coefficient (SE) = 0.917 (0.462), *p* = 0.048) were the predictors of desirable behavior.

**Conclusion:**

Overall, the UTAR community had demonstrated a good level of knowledge and preventive behaviors, albeit with some areas for improvement.

## Introduction

Coronavirus disease (COVID-19) poses a major health crisis globally (1). With the implementation of COVID-19 vaccination, it is expected that the risk of infection, morbidity and mortality will be reduced (2, 3). However, there is a significant disparity in the distribution of vaccines across countries (4, 5). This is further hampered by the slow uptake of the vaccine by the community (general public) in majority of countries due to uncertainty about side effects and complications following vaccination (6, 7). The continued surge of the infection that has been seen in numerous countries and regions is ascribed partly to the slow vaccination rate (8, 9) and partly to the emergence of COVID-19 variants at the community level (10). This is the apparent situation locally which is evidenced by the third wave with an unprecedented increase in the number of cases reported daily, and the increasing number of deaths (11). Similar dire situations are evident elsewhere in South Asia, South East Asia, South America and the African continent (11).

The challenges faced by the Malaysian authorities is how to fine-tune the balancing act of applying the movement control order (MCO) to restrict movement of people on the one hand, and allowing businesses to remain open to protect the economy on the other (12). Further, even with clear guidelines on standard operating procedures (SOPs) and implementation of different levels of MCO, the preventive measures such as wearing a face mask when leaving the home seems to be lacking locally (51.2%) (13), causing an unstable pattern of daily new cases (14).

In the university setting, staff and students alike face numerous challenges including the change in teaching and learning modes, implementation and observance of strict measures when required to be on campus, not to mention the lifestyle adjustments needed to mitigate the risk of COVID-19 infection and managing the resultant mental stress. How well the university community adjusts to this existential challenge is likely to be dependent on numerous individual and environmental factors.

Numerous studies conducted among the Asian population had pointed to some of these factors including knowledge, perception and practice in relation to COVID-19. Overall, with respect to the university community, the level of knowledge was found to be related to educational level (15), nature of courses pursued (15–17) and gender (17, 18). Further, it appeared that COVID-19 knowledge was positively correlated with risk perception and preventive behavior (19).

With respect to behavior, specifically pertaining to the use of preventive measures to minimize the risk of infection, the results are quite varied and concerning. For example, the use of face masks, an essential practice particularly in crowded places, has been reported to be <20% among university students in Pakistan (20), while that in Indonesia was quite high at 86.9% (17). Likewise, the findings for other key preventive measures such as hand hygiene (16, 20–22) and social distancing (18, 21, 23) are also quite variable.

A study from Indonesia reported that predictors of good preventive behavior were male gender and being a medical student (17) while another study from Egypt found that female gender and high perceived risk of infection were the predictors (18). However, risk perception was found to be inversely correlated with preventive measures among medical students in Iran (24) highlighting the differences in results from studies across the region. There are relatively few studies on COVID-19 risk perception among the Asian population, one of which recorded that 27.3% of Indian university students consider that they have a high probability of getting COVID-19 (22). In another study among Chinese university students, it was found that good knowledge, female gender and being non-medical students were predictor of high risk- perception for COVID-19 non-medical students and those with good knowledge in COVID-19 (25).

Based on the literature review, it is apparent that the predictors of knowledge on COVID-19 and the practice of preventive behavior among the university community is quite variable across the region. This could partly be due to difference in local culture across different nationalities as well as difference in the success or lack of in managing the infection in different countries. In addition, there is no single study that provides a comprehensive analysis on predictors of COVID-19 knowledge, preventive behavior, and risk perception. Nevertheless, with the available information, we hypothesize that the factors mentioned in the preceding paragraph, could have a significant association with COVID-19 knowledge, perception and behavior. Hence, this study provides an overall assessment of these parameters and their associated factors, in order to provide a clearer overview of the status among our university community.

## Materials and Methods

### Study Design and Study Population

This was a cross-sectional online survey conducted between 01 September 2020 and 28 February 2021. The study population for this study included all administrative staffs, academic staffs and students of Universiti Tunku Abdul Rahman (UTAR).

### Sampling Design and Sample Size Calculation

A convenience sampling was conducted for staffs and students at the UTAR Campus. The details of locality and the population (Number and percentage of staffs and students, number and percentage of administrative and academic staffs, and student breakdown by category are shown in Supplementary Tables S1, S2). Invitations to participate this online survey were sent *via* email; access to the questionnaire was only allowed if participants endorsed the informed consent. The email contacts were obtained with permission from the Human Resources Department and Student Affairs Offices.

Determination of the sample size was based on an estimated proportion of respondents with good knowledge of 80% (13), a margin of error (e) set at 5% and confidence interval at 95% (α = 0.05). The calculated sample size using the formula from Daniel and Cross (26) was 241.

### Instruments

This online survey consisted of several sections and was estimated to take 10–15 min to complete. We used the survey tool “Monitoring knowledge, risk perceptions, preventive behaviors and trust to inform pandemic outbreak response” prepared by World Health Organization (WHO) (27), with minor modifications to a few of the questions. The components of the WHO instrument that were employed were sociodemographic information, knowledge on COVID-19, perception of self-risk, preparedness and perceived self-efficacy, preventive measures and behavior (Supplementary Tables S3-S5).

### Study Variables and Scoring Method

The online questionnaire consisted of six five sections which are (i) sociodemographic information, (ii) knowledge on COVID-19, (iii) perceived self-risk as well as preparedness and self-efficacy, (iv) preventive measures, (v) behaviors- unwanted and desirable. The scoring details of each part are described below.

Socio-demographic information included age, gender, marital status, occupation, highest education obtained (for staffs) and level of study (for students), household size, living with dependents (children below 18 years old) and high-risk individuals (elderly above 60 years old). Additional items covered chronic medical illnesses, testing status for COVID-19 and the presence of positive COVID-19 cases within close social group(s).

Knowledge on COVID-19 was assessed using 22 questions (options given were “yes,” “no” and “don't know”); the correct response to each question was coded as a “1” and incorrect response was coded as a “0.” The total score for knowledge was calculated as the sum of the responses for the 22 questions, which yielded a total score ranging from 0 to 22. A higher score was indicative of a higher level of knowledge on COVID-19. The items in knowledge on COVID-19 were adapted from multiple sources (24, 28–32).

Perception of self-risk was explored using three questions covering probability of contracting the infection, susceptibility to the infection and the severity of the illness if infected. Preparedness and perceived self-efficacy comprised two questions which covered self-protection ability and disease-avoidance ability. Scoring was based on a 7-point Likert scale; the details of the options and the scores for each of these items, which are variable across items, are given in **Table 4**. In both cases the scores for the individual items were summed to give an aggregate score for statistical analysis.

Preventive (own) measures comprise 10 items to examine how much the respondents had actually done to protect themselves from getting COVID-19 (options given were “yes,” “no” and “does not apply”). The total score for this part ranged from 0 to 10; a higher score indicated better preventive behavior toward COVID-19. This set of questions was partly modified with the removal of a question on the use of antibiotics for COVID-19 prevention and a second question on avoiding social events. Instead, three other questions which asked about covering the mouth and nose when sneezing, use of caution when opening parcels and self-isolation when feeling unwell were added to better reflect the local situation and context.

The questionnaire on behavior of the respondents during the pandemic and the MCO comprised questions on both unwanted and desirable behaviors. The content included six items on unwanted behavior, as given in the WHO instrument, as well as three other questions on other behaviors deemed to be desirable/appropriate. The options and the scores were “Does not apply” (score 0), “I don't plan to do that” (score 1), “I plan to do that” (score 2) and “I already did that” (score 3). For unwanted behavior, the higher the score, the greater the inclination to indulge in unwanted behaviors; likewise, appropriate behaviors adopted during the pandemic were indicated by higher scores.

### Statistical Analysis

Data analysis was performed by using the statistical software package IBM SPSS Statistics Version 21.0 for Windows. All data were presented as mean ± standard de*via*tion or count, frequency (*n*, %), and ranges whenever appropriate. Independent *t*-test was used to compare the means of relevant variables between staffs and the students. The association between sociodemographic variables on one hand, and the total scores for knowledge and preventive (own) measures on the other, were tested with Chi-square test; Fisher's exact test was used if the assumptions of the Chi-square test were violated.

Simple linear regression was conducted to identify factors associated with knowledge, preventive measures adopted, self-risk perception, preparedness & perceived self-efficacy, and behavior. Variables with *p* < 0.25 were selected for further analysis using multiple linear regression to obtain adjusted B coefficients and their standard errors using the enter method. Variables with a *p* < 0.05 were considered statistically significant. In addition, we performed exploratory analysis to examine predictors of knowledge, preventive measures adopted, self-risk perception, preparedness & perceived self-efficacy, and behavior stratified by students and staffs; the results of simple and multiple linear regression for students are shown in Supplementary Table S6 and that for staffs in Supplementary Table S7.

Further analysis was performed to determine if COVID-9 risk perception, preparedness & perceived self-efficacy have mediating effects on the relationship between knowledge on COVID-19 and behaviors including unwanted and desirable behaviors as well as preventive (own) measures using the PROCESS macro in IBM SPSS (version 21). The indirect effect was considered as significant if the 95% confidence interval did not include 0 value (33).

## Results

A total of 434 responses were received comprising 93 (21.4%) from staff members and 341 (78.6%) from students. The characteristics of the respondents are presented in Table 1. The age among the staff is 36.6 ± 12.1 and that among students is 21.6 ± 2.1 (mean ± SD). Overall, females make up 58.4% of the respondents. Among the staffs, slightly under half (47.3%) are married, 78.5% have attained higher level education and a minority (6.5%) live alone. Among students, the majority (88.5%) are pursuing an undergraduate degree; all except 3 are single; the large majority (97.7%) live in households of 2 or more members.

**Table 1:** Socio-demographic characteristics of respondents (*n* = 434).

**Characteristics**	**Category**	**All (*n* = 434)**	**Staffs (*n* = 93)**	**Students (*n* = 341)**	***p*** **-values**
Age	Mean ± SD	24.8 ± 8.5	36.6 ± 12.1	21.6 ± 2.1	<0.001
	Median (IQR)	22 (2.3)	36 (17.5)	21 (3.0)	-
	Range	18–74	19–74	18–35	-
Sex	Male	172 (39.6)	30 (32.3)	142 (41.6)	0.101
	Female	262 (60.4)	63 (67.7)	199 (58.4)	
Marital status	Single or divorced	387 (89.2)	49 (50.7)	338 (99.1)	<0.001
	Married	47 (10.8)	44 (47.3)	3 (0.9)	
Role in the university	Administrative staffs	-	43 (26.2)	-	-
	Academic staffs	-	50 (73.8)	-	-
	Student	-	-	341 (100.0)	-
Highest education level obtained among	Secondary		20 (21.5)	-	-
administrative and academic staffs (*n* = 93)	Diploma/Bachelor degree		21 (22.6)	-	-
	Postgraduate/professional degree		52 (55.9)	-	-
Level of education among students	Foundation		-	31 (9.1)	-
(*n* = 341)	Undergraduate degree		-	302 (88.5)	-
	Postgraduate degree		-	8 (2.4)	-
Chronic illness	Don't know	22 (5.0)	4 (4.3)	18 (5.3)	0.224
	No	403 (92.9)	85 (91.4)	318 (93.3)	
	Yes	9 (2.1)	4 (4.3)	5 (1.4)	
Household size including the respondent	1	14 (3.2)	6 (6.5)	8 (2.3)	0.001
	2–4	229 (52.8)	61 (65.5)	168 (49.3)	
	5 or more	191 (44.0)	26 (28.0)	165 (48.4)	
Living with children (<18 years old)	No	282 (65.0)	53 (57.0)	229 (67.2)	0.069
	Yes	152 (35.0)	40 (43.0)	112 (32.8)	
Living with elderly (≥60 years old)	No	294 (67.7)	62 (66.7)	232 (68.0)	0.803
	Yes	140 (32.3)	31 (33.3)	109 (32.0)	
COVID testing and status	Not tested; status unknown	384 (88.5)	82 (88.2)	302 (88.6)	0.157
	Tested; status negative	49 (11.3)	10 (10.8)	39 (11.4)	
	Tested; status positive	1 (0.2)	1 (1.0)	0 (0.0)	
Immediate social contact who are or have been	No	314 (72.4)	72 (77.4)	242 (71.0)	0.218
infected with COVID-19	Yes	120 (27.6)	21 (22.6)	99 (29.0)	
Location of residence with regards to Ministry of	Don't know	41 (9.5)	4 (4.2)	37 (10.9)	0.240
Health Malaysia COVID-19 zoning	Green (areas without any active positive case)	29 (6.7)	5 (5.4)	24 (7.0)	
	Yellow (areas with 1 to 20 active positive cases)	44 (10.1)	10 (10.8)	34 (10.0)	
	Orange (areas with 21 to 40 active positive cases)	0 (0.0)	0 (0.0)	0 (0.0)	
	Red (areas with more than active 40 positive cases)	320 (73.7)	74 (79.6)	246 (72.1)	

*Data are presented as: mean ± standard deviation, median (interquartile range), range, count, frequency (%). SD = standard deviation; IQR = interquartile range*.

Overall, the large majority of the respondents do not have any chronic illness; With regards to living arrangement, 32.3% live with elderly members and 35% with dependents under age 18; 320 (73.7%) reside in high-risk or red zones. Lastly, the large majority are satisfied with the support services provided and are compliant with the public health measures recommended, albeit to varying degrees. The distribution of individual socio-demographic variables in both groups are statistical different in terms of age, marital status, and household size including the respondent (*p* < 0.05).

Table 2 is a summary of the outcome measurements. Overall, there is no statistical difference in the total score of knowledge on COVID-19, of self-risk perception, preparedness & perceived self-efficacy, preventive (own) measures, as well as unwanted and desirable behaviors between staffs and students. The descriptive analysis for each outcome measure is detailed in the subsequent sections. Subgroup analysis by gender for the following assessment was shown in (Supplementary Table S8).

**Table 2:** Summary of results on knowledge, risk perception, preparedness & perceived self-efficacy, preventive measures, and behavior related to COVID-19 (*n* = 434).

**Assessment (minimum and maximum score)**	**Category**	**All (*n* = 434)**	**Staffs (*n* = 93)**	**Students (*n* = 341)**	***p*** **-values**
**Knowledge on COVID-19** (0–22)	Mean ± SD	18.72 ± 2.73	19.04 ± 2.6	18.64 ± 2.77	0.204
	Median (IQR)	19.0 (4.0)	20.0 (3)	19.0 (4)	
	Range	8–22	9–22	8–22	
**COVID-19 risk perception**					
Aggregate score	Mean ± SD	10.27 ± 2.93	10.24 ± 3.03	10.27 ± 2.91	0.941
(3–21)	Median (IQR)	11.0 (4.0)	11.0 (4.0)	11.0 (3.0)	
	Range	3–18	3–17	3–18	
Probability of infection	Mean ± SD	3.38 ± 1.37	3.33 ± 1.28	3.39 ± 1.40	0.724
(1 = extremely unlikely; 7 = extremely likely)	Median (IQR)	3.0 (2)	3.0 (2.0)	3.0 (2.0)	
	Range	1–7	1–7	1–7	
Susceptibility to the disease	Mean ± SD	3.49 ± 1.37	3.44 ± 1.39	3.50 ± 1.37	0.707
(1 = not at all susceptible; 7 = very susceptible)	Median (IQR)	4.0 (2.0)	4.0 (2.0)	4.0 (2.0)	
	Range)	1–7	1–7	1.7	
Severity of illness	Mean ± SD	3.40 ± 1.49	3.47 ± 1.56	3.38 ± 1.47	0.598
(1 = very strongly disagree; 7 = very strongly agree)	Median (IQR)	3.0 (2.0)	4.0 (3.0)	3.0 (2.0)	
	Range)	1–7	1–7	1–7	
**Preparedness & perceived self-efficacy**					
Aggregate score	Mean ± SD	10.25 ± 1.73	10.20 ± 1.86	10.26 ± 1.69	0.779
(2–14)	Median (IQR)	10.0 (2.0)	10.0 (2.0)	10.0 (2.0)	
	Range)	5–14	5–14	5–14	
Protection ability	Mean ± SD	5.68 ± 0.99	5.74 ± 0.97	5.66 ± 1.0	0.480
(1 = not at all;	Median (IQR)	6.0 (1.0)	6.0 (1.0)	6.0 (1.0)	
7 = very much so)	Range)	1–7	3–7	1–7	
Avoidance ability (1 = extremely difficult; 7 = extremely easy)	Mean ± SD	4.57 ± 1.20	4.46 ± 1.32	4.60 ± 1.17	0.324
	Median (IQR)	5.0 (1.0)	5.0 (1.0)	5.0 (1.0)	
	Range	1–7	1–7	1–7	
**Preventive (own) measures**	Mean ± SD	8.71 ± 1.47	8.9 ± 1.18	8.66 ± 1.53	0.072
(0–10)	Median (IQR)	9.0 (2.0)	9 (2)	9 (2)	
	Range	4–10	5–10	4–10	
**Unwanted behaviors**	Mean ± SD	7.21 ± 3.33	7.47 ± 3.18	7.14 ± 3.37	0.398
(0–18)	Median (IQR)	7.0 (4.0)	8.0 (3.0)	7.0 (4.0)	
	Range)	0–18	2–18	0–18	
**Desirable behaviors**	Mean ± SS	5.63 ± 1.99	5.98 ± 1.94	5.54 ± 2.0	0.058
(0–9)	Median (IQR)	6.0 (3.0)	6.0 (3.50)	6.0 (2.0)	
	Range	0–9	2–9	0–9	

*Data are presented either in mean ± standard deviation (SD), median (interquartile range), range, frequency (%)*.

### Knowledge of COVID-19

The mean knowledge score was 18.7 points ± 2.7 (mean ± SD) out of a total of 22 points (Table 2). Majority of the respondents had good knowledge of COVID-19 for most of the items with the exceptions of less frequently encountered clinical features such as diarrhea, muscle/body aches, headache and runny/stuffy nose (Table 3). Noticeably, there was a misconception that using antibiotics is effective for preventing the spread of the infection among 47.9% of the respondents. It is worthwhile to report that close to 100% of the respondents were knowledgeable about preventive measure such as the use of face masks (item #18), physical distancing (item #20) and avoidance of touching the face with unwashed hands (item 14).

**Table 3:** Assessment of knowledge regarding COVID-19 (*n* = 434).

**Statement [Correct answer]**	**Correct answer, *N* (%)**	**Staffs,** ***N* (%)**	**Students,** ***N* (%)**
1. Fever can be a symptom of the novel coronavirus [yes]	406 (93.5)	89 (95.7)	317 (93.0)
2. Cough can be a symptom of the novel coronavirus [yes]	405 (93.3)	89 (95.7)	316 (92.7)
3. Shortness of breath can be a symptom of the novel coronavirus [yes]	398 (91.7)	90 (96.8)	308 (90.3)
4. Sore throat can be a symptom of the novel coronavirus [yes]	355 (81.8)	78 (83.9)	277 (81.2)
5. Runny or stuffy nose can be a symptom of the novel coronavirus [yes]	268 (61.8)	54 (58.1)	214 (62.8)
6. Muscle or body aches can be a symptom of the novel coronavirus [yes]	259 (59.7)	61 (65.6)	198 (58.1)
7. Headaches can be a symptom of the novel coronavirus [yes]	273 (62.9)	65 (69.9)	208 (61.0)
8. Fatigue can be a symptom of the novel coronavirus [yes]	360 (82.9)	79 (84.9)	281 (82.4)
9. Diarrhea can be a symptom of the novel coronavirus [yes]	230 (53.0)	55 (59.1)	175 (51.3)
10. Loss of taste and smell can be a symptom of the novel coronavirus [yes]	408 (94.0)	88 (94.6)	320 (93.8)
11. There is a vaccine for the COVID-19 infection [yes in 2021; No in 2020]	369 (85.0)	73 (78.5)	296 (86.8)
12. The Maximum incubation period of the novel coronavirus can be up to 14 days [yes]	401 (92.4)	88 (94.6)	313 (91.8)
13. Hand washing for at least 20 seconds is an effective measure to prevent the spread and infection of the novel coronavirus [yes]	416 (95.9)	89 (95.7)	327 (95.9)
14. Avoiding touching your eyes, nose, and mouth with unwashed hands is an effective measure to prevent the spread and infection of the novel coronavirus [yes]	428 (98.6)	91 (97.8)	337 (98.8)
15. Use of disinfectants to clean hands when soap and water was not available for washing hands is an effective measure to prevent the spread and infection of the novel coronavirus [yes]	407 (93.8)	88 (94.6)	319 (93.5)
16. Staying home when you were sick or when you had a cold is an effective measure to prevent the spread and infection of the novel coronavirus [yes]	423 (97.5)	92 (98.9)	331 (97.1)
17. Covering your mouth and nose when you cough or sneeze is an effective measure to prevent the spread and infection of the novel coronavirus [yes]	427 (98.4)	93 (100.0)	334 (97.9)
18. Wearing a face mask is an effective measure to prevent the spread and infection of the novel coronavirus [yes]	433 (99.8)	92 (98.9)	341 (100.0)
19. Using antibiotics is an effective measure to prevent the spread and infection of the novel coronavirus [no]	226 (52.1)	61 (65.6)	165 (48.4)
20. Physical distancing (keeping minimum 1 meter between you and other persons outside your house is an effective measure to prevent the spread and infection of the novel coronavirus [yes]	430 (99.1)	92 (98.9)	338 (99.1)
21. Self-isolation is an effective measure to prevent the spread and infection of the novel coronavirus [yes]	414 (95.4)	85 (91.4)	329 (96.5)
22. Disinfecting surfaces is an effective measure to prevent the spread and infection of the novel coronavirus [yes]	397 (91.5)	86 (92.5)	311 (91.2)

*Data are presented in frequency (%)*.

### COVID-19 Risk Perception, and Preparedness and Perceived Self-Efficacy

For risk perception, scores of 1–2 is categorized as minimal, 3–5 as mild to moderate and 6–7 as high. As shown in Table 4, most respondents considered themselves to be at minimal or mild to moderate risk for probability of getting infected, susceptibility to infection and severity of illness; only 6.7, 7.6, and 9.9% rated themselves to be at high risk respectively.

**Table 4:** COVID-19 Risk Perception, and Perceived Preparedness & Self-efficacy (*n* = 434).

**Perception statements**	**1**	**2**	**3**	**4**	**5**	**6**	**7**
**COVID-19 Risk Perception**							
1. What do you consider to be your own probability of getting infected with the novel coronavirus? (1 = extremely unlikely; 7 = extremely likely)	26 (6.0)	114 (26.3)	89 (20.5)	114 (26.3)	62 (14.3)	24 (5.5)	5 (1.2)
2. How susceptible do you consider yourself to an infection with the novel coronavirus? (1 = not at all susceptible; 7 = very susceptible)	25 (5.8)	99 (22.8)	85 (19.6)	128 (29.5)	64 (14.7)	27 (6.2)	6 (1.4)
3. I think I will have severe disease. (1=very strongly disagree; 7=very strongly agree)	42 (9.7)	99 (22.8)	78 (18.0)	125 (28.8)	47 (10.8)	34 (7.8)	9 (2.1)
**Preparedness and Perceived Self-efficacy**							
4. I know how to protect myself from coronavirus. (1 = not at all; 7 = very much so)	1 (0.2)	1 (0.2)	12 (2.8)	29 (6.7)	122 (28.1)	184 (42.4)	85 (19.6)
5. For me avoiding an infection with the novel coronavirus in the current situation. (1 = extremely difficult; 7 = extremely easy)	3 (0.7)	18 (4.1)	53 (12.2)	124 (28.6)	150 (34.6)	62 (14.3)	24 (5.5)

*Data are presented in frequency (%)*.

With respect to preparedness & perceived self-efficacy, a low score represents a low level and a high score reflects a high level of preparedness and self-efficacy. The average scores (mean ± SD) for the two individual questions, self-protection ability and avoidance ability, were 5.68 ± 0.99 and 4.57 ± 1.20 respectively. 62.0% of respondents considered that they have a high ability to protect themselves against the infection (score 6–7) whereas only 19.8% indicated a high level of ability to avoid the infection.

### Assessment of Preventive (Own) Measures Against COVID-19

Overall, over 80% (85.5–100%) of the respondents had exercised the standard preventive measures against COVID-19 (Table 5), the exception being opening letters or parcels (item #6; 68.2%), disinfecting surfaces (item #10; 75.1%) and self-isolation if unwell” (Item #9; 76.5%).

**Table 5:** Self-assessment of preventive measures taken against COVID-19 by genders (*n* = 434).

**Statements**	**Yes, *N* (%)**	**Male, *N* (%)**	**Female, *N* (%)**
1. Hand washing for at least 20 seconds	371 (85.5)	140 (81.4)	231 (88.2)
2. Avoid touching your eyes, nose, and mouth with unwashed hands	396 (91.2)	149 (86.6)	247 (94.3)
3. Use of disinfectants to clean hands when soap and water was not available for washing hands	401 (92.4)	154 (89.5)	247 (94.3)
4. Staying home when you were sick or when you had a cold	390 (89.9)	155 (90.1)	235 (89.7)
5. Covering your mouth and nose when you cough or sneeze	420 (96.8)	164 (95.3)	256 (97.7)
6. Using caution when opening letters/parcels	296 (68.2)	104 (60.5)	192 (73.3)
7. Wearing a face mask	434 (100)	172 (100.0)	262 (100.0)
8. Physical distancing (keeping minimum 1 meter between you and other persons outside your household)	416 (95.9)	163 (94.8)	253 (96.6)
9. Self-isolate when unwell	332 (76.5)	136 (79.1)	196 (74.8)
10. Disinfecting surfaces	326 (75.1)	119 (69.2)	207 (79.0)

*Data are presented in frequency (%). Male, n = 172; Female, n = 262*.

**Figure 1:**
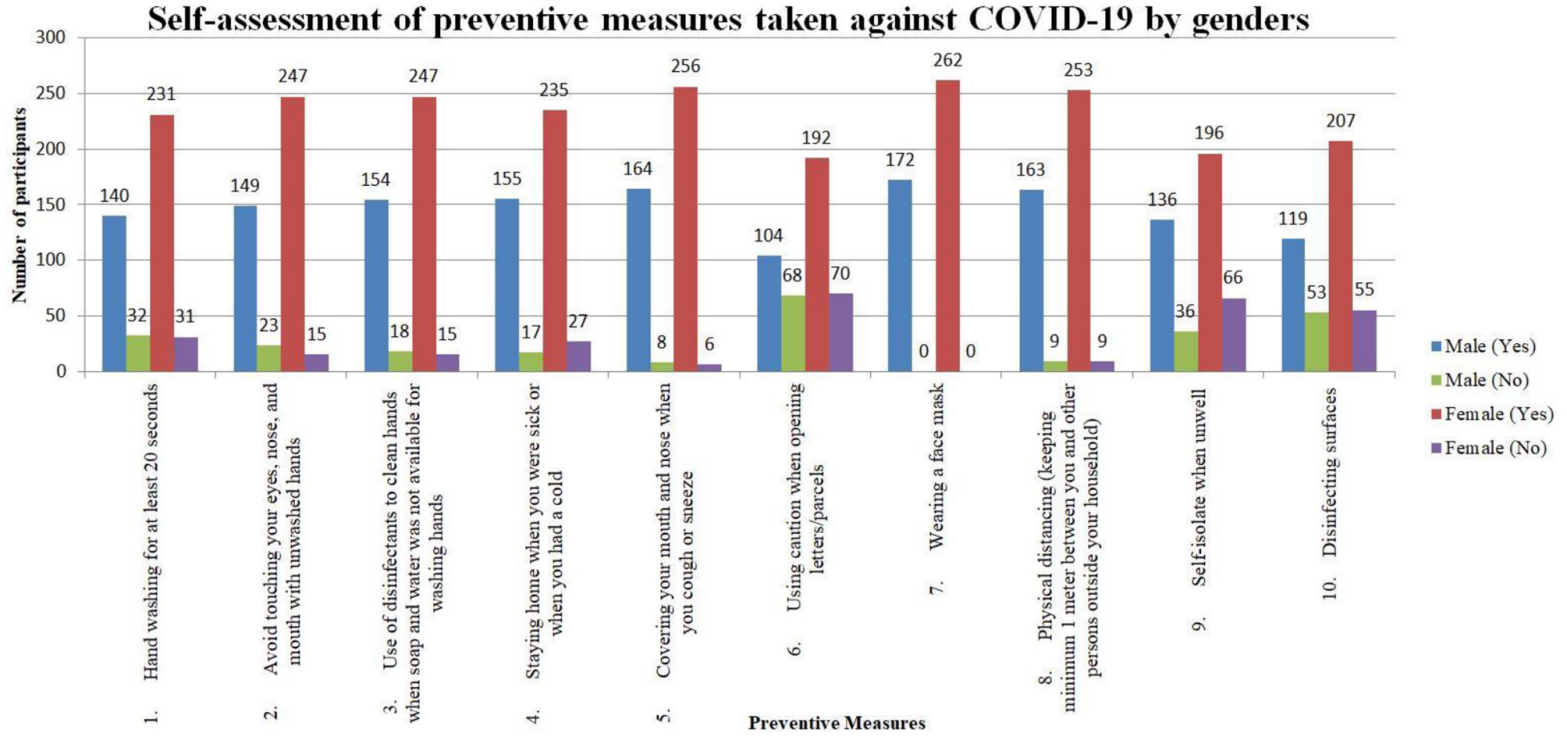
Self-assessment of preventive measures taken against COVID-19 by genders.

As shown in Figure 1, gender difference was seen in a few of the preventive (own) measures. Significant differences were present with regards to (i) “avoiding touching your eyes, nose, mouth with unwashed hands” where almost twice as many males (13.4%) did not practice this measure compared to females (5.7%) (with a *p*-value of 0.006; and (ii) “using caution when opening letters/parcels” where around one-third of males (39.5%) and 26.7% of females did not practice this measure (*p* = 0.005). A relatively high proportion of males (30.8%) and females (21.0%) did not practice “disinfecting surfaces.”

### Behavior During the Pandemic

A series of questions were designed to document the behavior of the respondents during the pandemic as well as the MCO introduced during the pandemic. They comprise 6 items covering unwanted behavior and 3 items on desirable behavior as shown in Table 6. The respondents were required to select the answers on a four options Likert scale. A notable observation in terms of unwanted behavior is the high proportion (79.0%) who plan to or have avoided people deemed to be high risk individuals (from regions where the infection rate is high). This was followed by decreased exercise (41.7%) and delay visiting the doctor for health issues that they consider can be postponed (34.1%). With regards to desirable behavior, the majority (96.5%) stated that they plan to or have purchased personal protection equipment such as masks and gloves, while 63.4% plan to or have requested their relatives not to visit and 27.2% decided not to allow their children to visit friends.

**Table 6:** Behaviors during the pandemic and the MCO (*n* = 434).

**Statements**	**Does not apply** **[Score = 0]**	**Do not plan to do so** **[Score = 1]**	**Plan to do so** **[Score = 2]**	**Have done so** **[Score = 3]**
**Unwanted behavior**				
1. Avoid people who come from countries/regions where daily infection rate of COVID-19 is high	65 (15.0)	26 (6.0)	90 (20.7)	253 (58.3)
2. Avoid going to the doctor with issues that could be postponed	143 (32.9)	143 (32.9)	69 (15.9)	79 (18.2)
3. Buy drugs that they have heard are good for treating COVID-19	197 (45.4)	207 (47.7)	12 (2.8)	18 (4.1)
4. Exercised less than I usually do	109 (25.1)	144 (33.2)	33 (7.6)	148 (34.1)
5. Drank more alcohol than I usually do	224 (51.6)	172 (39.6)	9 (2.1)	29 (6.7)
6. Ate more unhealthy food than I usually do	146 (33.6)	210 (48.4)	12 (2.8)	66 (15.2)
**Desirable behavior**				
7. Ask family members or friends not to visit	62 (14.3)	97 (22.4)	93 (21.4)	182 (41.9)
8. Decide that their child cannot meet with friends	260 (59.9)	56 (12.9)	41 (9.4)	77 (17.7)
9. Buy personal protection equipment	9 (2.1)	6 (1.4)	17 (3.9)	402 (92.6)

*Data are presented in frequency (%)*.

### Factors Associated With Knowledge and Preventive (Own) Measures for COVID-19

Based on multiple linear regression analysis (Table 7), factors associated with higher knowledge score were older age (*p* = 0.039), those from medical faculty (p=0.039) and residence located in red (high risk) zones (*p* = 0.005). With regards to preventive (own) measures, it was found that higher knowledge score (*p* <0.001) and female gender (*p*- = 0.014) were significantly associated with higher preventive measure scores.

**Table 7:** Factors associated with knowledge and preventive (own) measures toward COVID-19 in respondents (*n* = 434).

**Variables**	**Knowledge of COVID-19**				**Preventive (own) measures**			
	**Simple linear regression Crude B coefficient (S.E.)**	***p*** **-value**	**Multiple linear regression Adjusted B coefficient (S.E)**	***p*** **-value**	**Simple linear regression Crude B coefficient (S.E)**	***p*** **-value**	**Multiple linear regression Adjusted B coefficient (S.E)**	***p*** **-value**
Knowledge of COVID-19	-	-	-	-	0.127 (0.025)	0.000	0.116 (0.025)	**<0.001**
Age	0.034 (0.015)	0.026	0.046 (0.022)	**0.039**	0.010 (0.008)	0.223	−0.004 (0.012)	0.708
Female (Reference - males)	0.457 (0.268)	0.089	0.345 (0.267)	0.197	0.413 (0.143)	0.004	0.348 (0.142)	**0.014**
Married (Reference - Single and divorced)	0.477 (0.422)	0.259	-	-	0.225 (0.226)	0.321	-	-
Staff (Reference - students)	0.407 (0.320)	0.204	−0.319 (0.459)	0.487	0.268 (0.171)	0.118	0.223 (0.242)	0.357
Medical Faculty (Reference - Non-Medical Faculty)	1.050 (0.420)	0.013	0.870 (0.420)	**0.039**	0.344 (0.226)	0.129	0.124 (0.224)	0.581
With chronic medical illness (Reference - no chronic medical illness)	0.736 (0.922)	0.425	-	-	0.746 (0.493)	0.131	0.684 (0.489)	0.163
Tested and status positive (Reference - not tested, status unknown; tested, status negative)	1.279 (2.741)	0.641	-	-	1.289 (1.468)	0.381	-	-
Positive case(s) within social group (Reference - no positive cases)	0.140 (0.294)	0.633	-	-	−0.181 (0.157)	0.250	-	-
Red zone (Reference - other than red zone)	0.815 (0.296)	0.006	0.831 (0.295)	**0.005**	−0.102 (0.160)	0.524	-	-
Stay alone (Reference – not staying alone)	0.655 (0.743)	0.379	-	-	0.148 (0.399)	0.711	-	-
Household with children (Reference - without children)	−0.111 (0.275)	0.687	-	-	0.095 (0.148)	0.518	-	-
Household with elderly (Reference - without elderly)	−0.077 (0.281)	0.785	-	-	−0.095 (0.151)	0.529	-	-
Model intercept			16.758 (0.581)				6.367 (0.524)	

*The bold values indicate the p value < 0.05*.

The mediating effect of COVID-19 risk perception and of preparedness & perceived self-efficacy on the association between knowledge and preventive measures was explored. The analysis showed that both variables did not exert any significant mediating effect on the said relationship [indirect effect of risk perception was <0.001 (95% CI <0.001, <0.001); indirect effect of preparedness & perceived self-efficacy was 0.0002 (95% CI −0.006, 0.006)].

We further explored the predictors of knowledge and preventive measures toward COVID-19 stratified by student and staff. The results showed that among students, the predictor for higher score of knowledge of COVID-19 were being from medical faculty [B (SE) = 1.239 (0.490), *p* = 0.012] and residence located in red zone (high risk) [B (SE) = 0.900 (0.326), *p* = 0.006. However, no factors were found to be predictive of the level of COVID-19 knowledge among the staff.

With regards to preventive (own) measures, the predictors among students included knowledge of COVID-19 [B (SE) = 0.113 (0.029), *p* < 0.001] and being a female [B (SE) = 0.405 (0.165), *p* = 0.014, while that among staff members were knowledge of COVID-19 score [B (SE) = 0.097 (0.044), *p* = 0.030], those not from medical faculty [B (SE) = 0.731 (0.347), *p* = 0.038] and residence not located in red zone [B (SE)= −0.592 (0.284), *p* = 0.040.

### Factors Associated With COVID-19 Risk Perception and With Preparedness and Perceived Self-Efficacy

Results of multiple linear regression analysis (Table 8) demonstrate that predictors of COVID-19 risk perception were presence of positive COVID-19 cases among social contacts (*p* = 0.015) and living with elderly (*p* < 0.001). Preparedness and perceived self-efficacy were positively associated with presence of children in the household (*p* = 0.011) and negatively associated with the presence of COVID-19 cases among social contacts (*p* = 0.023).

**Table 8:** Factors associated with risk perception and with preparedness & perceived self-efficacy (*n* = 434).

**Variables**	**COVID-19 risk perception** **(Aggregate score)**				**Preparedness and perceived self-efficacy** **(Aggregate score)**			
	**Simple linear regression Crude B coefficient (S.E.)**	***p*** **-value**	**Multiple linear regression Adjusted B coefficient (S.E)**	***p*** **-value**	**Simple linear regression Crude B coefficient (S.E.)**	***p*** **-value**	**Multiple linear regression Adjusted B coefficient (S.E)**	***p*** **-value**
Knowledge of COVID-19	0.038 (0.052)	0.461	-	-	0.002 (0.030)	0.961	-	-
Preventive action	−0.005 (0.096)	0.957	-	-	0.119 (0.056)	0.035	0.100 (0.056)	0.075
Age	−0.007 (0.017)	0.684	-	-	0.003 (0.010)	0.755	-	-
Female (Reference - males)	−0.414 (0.287)	0.150	0.341 (0.283)	0.229	−0.146 (0.170)	0.389	-	-
Married (Reference - single and divorce)	−0.300 (0.453)	0.509	-	-	0.127 (0.267)	0.636	-	-
Staff (Reference -students)	−0.025 (0.343)	0.941	-	-	−0.057 (0.202)	0.779	-	-
With medical illness (Reference - no medical illness)	−0.386 (0.988)	0.696	-	-	0.881 (0.581)	0.131	0.863 (0.574)	0.133
Medical Faculty (Reference – Non-Medical Faculty)	−0.228 (0.453)	0.615	-	-	0.246 (0.267)	0.358	-	-
Tested and status positive (Reference - not tested, status unknown; tested, status negative)	3.741 (2.933)	0.203	2.972 (2.883)	0.303	1.755 (1.730)	0.311	-	-
COVID-19 positive case(s) within social group (Reference - no positive cases)	0.679 (0.313)	0.031	0.751 (0.308)	**0.015**	−0.459 (0.184)	0.013	−0.418 (0.183)	**0.023**
Red zone (Reference – not in red zone)	0.184 (0.320)	0.565	-	-	−0.150 (0.189)	0.426	-	-
Stay alone (Reference - not staying alone)	−0.867 (0.796)	0.277	-	-	0.629 (0.469)	0.181	0.765 (0.466)	0.102
With children in household (Reference – no children in household)	−0.199 (0.295)	0.501	-	-	0.427 (0.173)	0.014	0.440 (0.173)	**0.011**
Living with elderly (Reference - not living with elderly)	1.155 (0.296)	<0.001	1.137 (0.296)	**<0.001**	−0.146 (0.178)	0.412	-	-
Model intercept			9.617 (0.872)				9.298 (0.501)	

*The bold values indicate the p value <0.05*.

Exploratory analysis stratified by student and staff showed that among the students, the only predictor of COVID-19 high risk perception was living with elderly [B (SE) = 1.146 (0.334), *p* = 0.001] whereas among staffs, the only predictor of high-risk perception was living in households without children [B (SE) = 1.641 (0.614), *p* = 0.009].

Predictors for preparedness & self-efficacy among students include the absence of positive case among social contacts [B (SE) = 0.527 (0.198), *p* = 0.008] and living in households with children [B (SE) = 0.432 (0.192), *p* = 0.025]. In the case of staff, the predictors were being married [B (SE) = 2.421 (0.962), *p* = 0.012], absence of positive cases within their social group [B (SE) = 0.524 (0.198), *p* = 0.008] and living with children [B (SE) = 0.424 (0.191), *p* = 0.027].

### Factors Associated With Behavior During the COVID-19 Pandemic and the MCO

Following multiple linear regression analysis (Table 9), predictors of unwanted behavior during the COVID-19 pandemic were male gender (*p* = 0.039) and COVID-19 positive status (*p* = 0.003). For desirable behavior, the predictors were knowledge on COVID-19 (*p* = 0.048) and being married (*p* = 0.048).

**Table 9:** Factors associated with behavior during the pandemic and MCO (*n* = 434).

**Variables**	**Unwanted behavior**				**Desirable behavior**			
	**Simple linear regression Crude B coefficient (S.E.)**	***p*** **-values**	**Multiple linear regression Adjusted B coefficient (S.E)**	***p*** **-values**	**Simple linear regression Crude B coefficient (S.E)**	***p*** **-values**	**Multiple linear regression Adjusted B coefficient (S.E)**	***p*** **-values**
Knowledge of COVID-19	0.050 (0.059)	0.394	-	-	0.071 (0.035)	0.042	0.069 (0.035)	**0.048**
Risk perception score	0.021 (0.055)	0.701	-	-	0.008 (0.033)	0.807	-	-
Preparedness & perceived self-efficacy score	0.208 (0.092)	0.025	0.179 (0.091)	0.051	0.054 (0.055)	0.330	-	-
Age	0.030 (0.019)	0.114	0.020 (0.019)	0.289	0.019 (0.011)	0.098	−0.021 (0.019)	0.274
Female (Reference: Male)	−0.762 (0.325)	0.019	−0.664 (0.321)	**0.039**	0.112 (0.195)	0.568	-	-
Married Reference: Single/divorced)	0.523 (0.514)	0.309	-	-	0.819 (0.305)	0.008	0.917 (0.462)	**0.048**
Staff (Reference: Students)	0.329 (0.390)	0.398	-	-	0.442 (0.232)	0.058	0.217 (0.340)	0.524
Medical Faculty (Reference – Non-Medical Faculty)	−0.574 (0.514)	0.264	-	-	−0.135 (0.308)	0.660	-	-
With medical illness (Reference: None)	1.597 (1.120)	0.155	1.149 (1.120)	0.306	0.830 (0.670)	0.216	0.640 (0.676)	0.344
Tested and status positive (Reference: Not tested, tested negative)	10.811 (3.295)	0.001	9.736 (3.297)	**0.003**	3.376 (1.988)	0.090	3.694 (2.004)	0.066
Positive case in social group (Reference: None)	0.049 (0.358)	0.890	-	-	−0.032 (0.214)	0.882	-	-
Red zone (Reference: Non-red red zone)	−0.126 (0.363)	0.729	-	-	0.095 (0.217)	0.663	-	-
Staying alone (Reference: Staying with others)	0.738 (0.905)	0.415	-	-	0.307 (0.541)	0.571	-	-
Household with children (Reference: Without children)	−0.249 (0.335)	0.458	-	-	0.274 (0.200)	0.172	0.186 (0.203)	0.352
Household with Elderly (Reference: No elderly)	0.221 (0.342)	0.518	-	-	−0.183 (0.204)	0.370	-	-
Model intercept			5.243 (1.076)				4.622 (0.749)	

*The bold values indicate the p value <0.05*.

Analysis of the mediating effect of COVID-19 risk perception and of preparedness & perceived self-efficacy on the association between knowledge and behaviors (unwanted and desirable) showed that both variables did not exert any significant mediating effect on the relationships [Indirect effect of risk perception between knowledge and unwanted behavior was <0.001 (95% CI −0.01, 0.01); indirect effect of preparedness & perceived self-efficacy between knowledge and unwanted behavior was <0.001 (95% CI −0.013, 0.016); indirect effect of risk perception between knowledge and desirable behavior was 0.001 (95% CI −0.001, 0.01); indirect effect of preparedness & perceived self-efficacy between knowledge and desirable behavior was <0.001 (95% CI −0.005, 0.006).

Analysis stratified by student and staff indicated that among students, none of the factors appeared to be associated with either unwanted behavior or desirable behavior. Among staff, testing positive for COVID-19 [B (SE) = 8.911 (3.099), *p* = 0.005] was the only predictor of unwanted behavior, while living in households with children [B (SE) = 0.986 (0.434), *p* = 0.026] was the only predictor of desirable behavior.

## Discussion

The crisis caused by the COVID-19 virus has far-reaching effects in the field of higher education. In Malaysia, schools and institutes of higher learning were closed for varying duration during the first and second waves of the pandemic, with short breaks in between, spanning a period of almost 12 months at the time of this survey. This necessitated the use of on-line modes for communication, meetings and teaching-learning activities. All categories of staffs and all students were required to adapt fast to this change. During the short periods of relaxation of the movement restriction between the two waves, staffs and students were allowed on campus, in smaller numbers, with strict observation of public health measures to minimize the risk of infection. Hence, it is very important that everyone is knowledgeable about the infection and familiar with the preventive measures. This survey was conducted with the objective of determining the level of knowledge, the preventive measures practiced, COVID-19 risk perception, preparedness and perceived self-efficacy, and behavior among staffs and students, and their inter-relationships.

We found that the level of knowledge on COVID-19 was quite high among the respondents, with an average score of 18.7/22 (85.1%). While the knowledge score was good overall, there were some gaps with respect to the less frequent symptoms of COVID-19. More concerning is the misconception that antibiotics could be an effective way to prevent COVID-19 among 47.9% of the respondents, a finding similar to that found in a study which reported that as high as 66.4% of Australians believe that taking antibiotics regularly could possibly prevent COVID-19 (34). The proliferation of information over social media, including misinformation, could be a factor in this observation as well as in promoting risky behavior (35, 36).

The predictors of higher knowledge score included older age and residence within red (high-risk) zones. The association between older age and knowledge score is not surprising within the context of our study, as over half of the staff, who are obviously older than students, have postgraduate and/or professional degrees. The positive association between age and knowledge is corroborated by several studies (37–40). Further, Olaimet et al. (15) and Habatu et al. (16) have demonstrated that students engaged in postgraduate studies scored significantly higher compared to undergraduate students, providing support to the plausible association between educational level and knowledge. The association with location of residence can be understood from the perspective of the increased risk of infection if the residence is within a zone of high transmissibility of the SARS-CoV-2; hence the existential risk and the need to be more vigilant. This is likely to lead to more attention being paid to information related to the infection and its spread.

Other factors that have been reported to be associated with the level of knowledge include gender (16–18), and specifically in the case of students, the nature of the course taken (15–17). The association with gender is unclear as reported results are quite varied. We did not find a significant association between gender and knowledge similar to study by Olaimet et al. (15). We did, however, find significant association between knowledge and being in the medical course among the students, similar to the results from the above quoted studies.

The use of preventive measures against COVID-109 spread was relatively high among our respondents (overall score of 8.71/10). Specifically with regards to the use of face masks, the score was indeed 100%. In comparison, in an earlier study on the local population, the use of face masks was only 51.2% (13). This difference could be due to various factors, the most important being the fact that the quoted study was done in the early phase of the local outbreak, at which point in time, the reported daily number of cases were much smaller and presumably, the perceived risk of infection lower among the people, not forgetting the fact that face masks were not mandatory then. Data on the use of face masks as a preventive measure from other countries is also rather varied ranging from under 20% (20) to over 85% (17). Factors, other than that already mentioned, such as socio-economic and cultural difference could also account for such diversity of the reported data.

We found a significant association between good preventive behaviors and good level of knowledge. It is logical to assume that good knowledge of the disease transmission would promote the use of preventive measures; hence, the finding of similar results in several other studies (41–44). The other predictor for better preventive measure score was female gender, which was also seen in previous studies conducted in China, Turkey and Saudi Arabia (41, 45, 46). Such a finding could indicate that females tend to be more concerned about the infection and thus more likely to comply with safety measures (47). A meta-analysis published in 2016 in the context of respiratory epidemics such as influenza, found that females are more likely to practice non-pharmacological protective behavior than men (48).

It may be relevant to note that certain precautionary measures, including exercising caution when opening parcels and disinfecting surfaces, are practiced less frequently among the respondents. However, there is no consensus regarding the transmission of coronavirus through inanimate surfaces. Some studies have shown that the SARS-CoV-2 was detectable on plastic up to 5 days, and on paper between 3 h to 5 days; the variation of survivability of the coronavirus may be due to different environmental conditions such as temperature or humidity (49, 50). However, it was pointed out that the chance of transmission through such means is high only if an infected person coughs or sneeze on the surfaces and someone else touches that surface soon after that (within 1–2 h), thereby making this mode of transmission less likely (51).

Self-risk perception was analyzed from three aspects, the probability of being infected, the susceptibility to infection and the severity of illness if infected. Overall, the perception of high risk for disease contagion, susceptibility and severity was relatively low at 6.7, 7.6, and 9.9% respectively. In early 2020, an investigation of the risk perception among the public across several countries in Europe, United States of America and Asia was carried out by Dryhurst et al. (52). It was reported that the perceived risk, which varied from country to country, was nevertheless relatively high. The authors highlighted that overall, personal experience with the virus, prosocial values, and amplification about the infection through family and social contacts significantly influenced risk perception in more than half the countries studied. Although the risk perception among the participants in this study is comparatively low, the notable finding of a positive association between risk perception and presence of COVID-19 cases within social contacts, was similar to the observation of Dryhurst S et.al. In this paper, it was also pointed out that risk perception correlated significantly with reported adoption of preventative health behaviors in all the countries studied. We did not, however, find such an association. Another finding in our study is a positive association of higher self-risk perception with having elderly family members, which is not unexpected as older people who are at much higher risk of being infected could pose an increased risk to the entire family.

A negative association between perception of high self-risk and chronic medical illness was observed in our respondents. However, it is noted that in our study the number of participants with chronic illness were very small, which may negate the relevance of this finding. We note that the effect of medical comorbidities on risk perception has been mentioned in studies by Yan et al. (53), He et al. (54) and Laires et al. (55). In these studies, contrary to our observation, a positive association was found between chronic illness and risk perception. This is not unexpected in view of the widespread dissemination, through various media, about the increased risk for COVID-19 among people with chronic illnesses. Lastly, we also did not find any association between gender and self-risk perception in contrast to results from other studies (56, 57).

Preparedness and perceived self-efficacy were positively associated with the living in households with children and negatively associated with the presence of COVID-19 cases among social contacts. It is generally believed that children are at lower risk of infection compared to adults, with reported prevalence ranging from 1.2% in Italy (58) to 2% in China (59). Further, they are at much lower odds of being infected (pooled odds ratio = 0.56, 95% CI = 0.37–0.85) compared to adults (60). Nevertheless, owing to the relative lack of information on the long-term effects of COVID-19, it has been recommended that children should not be exposed to people outside the household and any individual who is unwell (61), highlighting the risk, albeit not as high as among adults. The negative association with the presence of COVID-9 among social contacts is rather more difficult to explain, as the opposite would seem to be more likely.

Overall, unwanted behavior was found to be significantly associated with the male gender and COVID-19 positive status. Knowledge on COVID-19 and being married were the predictors of desirable behaviors, a finding that is consistent with another study (62). This is not unexpected as married people, especially those with children and/or are staying with elderly parents would more likely adopt better preventive measures and behavior, within the context of local cultural norms – being more prosocial than individualistic.

Out of the six items on unwanted behavior, three were about lifestyle which included physical activity, alcohol consumption and unhealthy dietary intake. The fourth was on stereotyping people who are considered to be high risk individuals and the remaining two were on health literacy. In terms of lifestyle, males apparently tended to adopt more unwanted behavior, in particular with respect to diet and alcohol consumption (63). In the present study, we observe that males exercised less (*p* = ≤ 0.001) and consumed alcohol more (*p* < 0.016); however, the difference in terms of unhealthy diet was not significant (*p* = 0.117). With respect to stereotyping, we found that females tended to do this more (*p* = 0.005). In a study on Malaysians, Jaafar et al. reported that females have better sufficiency in health literacy than their male counterparts (64), a result that was consistent with the finding from other countries (65–68). We did not, however, observe this in the present study.

Regarding the confirmed diagnosis of COVID-19 as one of the predictors for unwanted behaviors, we note that there was only one person with a history of COVID-19 infection, a 44 years old male, with no history of chronic medical illness, lived in the green zone and with no known COVID-19 among his immediate social contacts. This person had affirmed that he had practiced all the unwanted behaviors.

The findings of this research could inform relevant management staff and policy makers of the university with regards to the gaps in knowledge, the perceptions and behavior of the staff and students related to the COVID-19 pandemic, thereby allowing adjustments to existing measures and policies with the aim to improve upon them if deemed necessary. As the pandemic is still evolving locally, and transmission has accelerated with the appearance of highly infections virus variants, measures must be continually monitored and adjusted with the changing landscape. While the results from this study may not be generalizable, it nevertheless provides a glimpse into the effects of the pandemic on a typical university community and provides a reference point for future studies.

The limitation of this study lies in the fact that it only involved the university community, and only one university. In addition, due to low response rate and sampling method used, sampling bias or non-response bias is expected. Therefore, the findings of this study cannot be generalized to the whole Universiti Tunku Abdul Rahman community or to university communities at large. Further, being a cross-sectional and largely descriptive study, no causal associations can be drawn from the results. Due to the dynamic nature of the pandemic, these results should be taken in context.

## Conclusion

COVID-19 pandemic is a major challenge to all aspects of living. This study showed that the majority of the university community had a good level of knowledge achieving an overall knowledge score of 18.7 per out of 22. Likewise, preventive action with an overall score of 8.7 out of 10. The perceived self-risk was relatively low implying that the respondents considered themselves to be quite safe. This could be related to their high level of preventive action. However, a low level of risk perception could lead to lowering the guard against the infection. Hence the needs for constant and timely update to inform and remind *via* social media, television and radio regarding the deadly nature of the infection is essential to aid in efforts to fight COVID-19 despite the introduction of vaccination.

## Data Availability Statement

Raw data supporting the conclusion can be obtained from corresponding author on special request.

## Ethics Statement

The studies involving human participants were reviewed and approved by Universiti Tunku Abdul Rahamn. The patients/participants provided their written informed consent to participate in this study.

## Author Contributions

KWL, SFY, HTO, PPL, NMH, and MSL: conceptualization, investigation, and methodology. MSL: data curation. KWL, SFY, and MSL: formal analysis. SFY: funding acquisition. HTO, PPL, and NMH: project administration and resources. KWL: software. SFY and MSL: supervision. HTO, PPL, and MSL: validation. KWL: visualization. KWL, SFY, and MSL: writing—original draft and writing—review and editing. All authors contributed to the article and approved the submitted version.

## Funding

This research was funded by Universiti Tunku Abdul Rahman, Grant Number: IPSR/RMC/UTARRF/2020-C2/Y01.

## Conflict of Interest

The authors declare that the research was conducted in the absence of any commercial or financial relationships that could be construed as a potential conflict of interest.

## Publisher's Note

All claims expressed in this article are solely those of the authors and do not necessarily represent those of their affiliated organizations, or those of the publisher, the editors and the reviewers. Any product that may be evaluated in this article, or claim that may be made by its manufacturer, is not guaranteed or endorsed by the publisher.
