# Supplementary material for: Knowledge, Perceptions and Behaviors Related to COVID-19 in a University Setting in Malaysia
Source: Front Public Health. 2022 Apr 11;10:873022. doi: 10.3389/fpubh.2022.873022 (PMC9035815; doi:10.3389/fpubh.2022.873022)
Supplement: Supplementary file 1 [file Data_Sheet_1.pdf]

| Supplementary Table S1: Details of number and percentage of staffs breakdown by category (n=1131) | | | | |
|---|---|---|---|---|
| No | Category | Population | Number of participants | % out of actual population |
| 1 | Administration Department (Administrative) | 414 | 43 | 10.4 |
| 2 | Faculty of Medicine and Health Sciences (Academic) | 137 | 12 | 8.7 |
| 3 | Faculty of Engineering (Academic) | 267 | 13 | 4.9 |
| 3 | Non-medical /non-engineering sciences (Academic) | 313 | 25 | 8.0 |
| | Total | 1131 | 93 | 8.2 |

| Supplementary Table S2: Details of number and percentage of students breakdown by category (n=10410) | | | | |
|---|---|---|---|---|
| No | Category | Population | Number of participants | % out of actual population |
| 4 | Student – Medicine and Health Sciences | 633 | 35 | 5.5 |
| 5 | Student – Engineering + Foundation in Sciences | 5025 | 180 | 3.6 |
| 6 | Student - Non-Medic/Engineering + Foundation in Art | 4752 | 126 | 2.7 |
| | Total | 10410 | 341 | 3.3 |

| Supplementary Table S3: Assessment of knowledge regarding COVID-19 | |
|---|---|
| Statement [Correct answer] | Options |
| 1. Fever can be a symptom of the novel coronavirus [yes] | • Yes<br>• No<br>• Don't know |
| 2. Cough can be a symptom of the novel coronavirus [yes] | |
| 3. Shortness of breath can be a symptom of the novel coronavirus [yes] | |
| 4. Sore throat can be a symptom of the novel coronavirus [yes] | |
| 5. Runny or stuffy nose can be a symptom of the novel coronavirus [yes] | |
| 6. Muscle or body aches can be a symptom of the novel coronavirus [yes] | |
| 7. Headaches can be a symptom of the novel coronavirus [yes] | |
| 8. Fatigue can be a symptom of the novel coronavirus [yes] | |
| 9. Diarrhea can be a symptom of the novel coronavirus [yes] | |
| 10. Loss of taste and smell can be a symptom of the novel coronavirus [yes] | |
| 11. Which statement is correct? [There is a vaccine for the COVID-19 infection ] | • There is a cure for the COVID-19 infection<br>• There is a vaccine for the COVID-19 infection<br>• There is currently no curative drugs or vaccine for the COVID-19 infection<br>• Don't know |
| 12. What is the maximum incubation period (i.e. the time from viral infection to developing symptoms of illness) of the novel coronavirus? | • Up to 3 days<br>• Up to 7 days<br>• Up to 14 days<br>• Don't know |
| 13. Hand washing for at least 20 seconds is an effective measure to prevent the spread and infection of the novel coronavirus [yes] | • Yes<br>• No<br>• Don't know |
| 14. Avoiding touching your eyes, nose, and mouth with unwashed hands is an effective measure to prevent the spread and infection of the novel coronavirus [yes] | |
| 15. Use of disinfectants to clean hands when soap and water was not available for washing hands is an effective measure to prevent the spread and infection of the novel coronavirus [yes] | |
| 16. Staying home when you were sick or when you had a cold is an effective measure to prevent the spread and infection of the novel coronavirus [yes] | |
| 17. Covering your mouth and nose when you cough or sneeze is an effective measure to prevent the spread and infection of the novel coronavirus [yes] | |
| 18. Wearing a face mask is an effective measure to prevent the spread and infection of the novel coronavirus [yes] | |
| 19. Using antibiotics is an effective measure to prevent the spread and infection of the novel coronavirus [no] | |
| 20. Physical distancing (keeping minimum 1 meter between you and other persons outside your house is an effective measure to prevent the spread and infection of the novel coronavirus [yes] | |

| | |
|---|---|
| 21. Self-isolation is an effective measure to prevent the spread and infection of the novel coronavirus [yes] | |
| 22. Disinfecting surfaces is an effective measure to prevent the spread and infection of the novel coronavirus [yes] | |

| Supplementary Table S4: Self-assessment of preventive measures taken against COVID-19 | |
|---|---|
| Which of the following measures have you taken to prevent infection from the novel coronavirus? Please indicate for all measures below whether you have already taken them | Options |
| 1. Hand washing for at least 20 seconds | • Yes<br>• No<br>• Does not apply |
| 2. Avoid touching your eyes, nose, and mouth with unwashed hands | |
| 3. Use of disinfectants to clean hands when soap and water was not available for washing hands | |
| 4. Staying home when you were sick or when you had a cold | |
| 5. Covering your mouth and nose when you cough or sneeze | |
| 6. Using caution when opening letters/parcels | |
| 7. Wearing a face mask | |
| 8. Physical distancing (keeping minimum 1 meter between you and other persons outside your household) | |
| 9. Self-isolate when unwell | |
| 10. Disinfecting surfaces | |

| Supplementary Table S5: COVID-19 Risk Perception, preparedness & Perceived self-efficacy, and behaviours during the pandemic and the MCO. | | |
|---|---|---|
| Tools | Statement | Scoring scheme |
| COVID-19 Risk Perception | 1. What do you consider to be your own probability of getting infected with the novel coronavirus? | 1-Extremely unlikely<br>7-Extremely likely |
| | 2. How susceptible do you consider yourself to an infection with the novel coronavirus? | 1-Not at all susceptible<br>7-Very susceptible |
| | 3. I think I will have severe disease | 1-very strongly disagree<br>7-very strongly agree |
| Preparedness and Perceived Self-efficacy | 1. I know how to protect myself from coronavirus | 1-Not at all<br>7-Very much so |
| | 2. For me avoiding an infection with the novel coronavirus in the current situation is … | 1-Extremely difficult<br>7-Extremely easy |
| Behaviour during the pandemic and the MCO (Unwanted behaviour) | 1. Avoid people who come from countries/regions where daily infection rate of COVID-19 is high | 0-Does not apply<br>1-Do not plan to do so<br>2-Plan to do so<br>3-Have done so |
| | 2. Avoid going to the doctor with issues that could be postponed | |
| | 3. Buy drugs that they have heard are good for treating COVID-19 | |
| | 4. Exercised less than I usually do | |
| | 5. Drank more alcohol than I usually do | |
| | 6. Ate more unhealthy food than I usually do | |
| Behaviour during the pandemic and the MCO (Desirable behaviour) | 1. Ask family members or friends not to visit | |
| | 2. Decide that their child cannot meet with friends | |
| | 3. Buy personal protection equipment | |

**Supplementary Table S6: Factors associated with knowledge and preventive (own) measures towards COVID-19 in students (n = 341).**

| Variables | Knowledge of COVID-19 | | | | Preventive (own) measures | | | | COVID-19 risk perception (Aggregate score) | | | | Preparedness & perceived self-efficacy (Aggregate score) | | | | Unwanted behaviour | | | | Desirable behaviour | | | |
|---|---|---|---|---|---|---|---|---|---|---|---|---|---|---|---|---|---|---|---|---|---|---|---|---|
| | Simple linear regression Crude B coefficient (S.E.) | p-value | Multiple linear regression Adjusted B coefficient (S.E) | p-value | Simple linear regression Crude B coefficient (S.E) | p-value | Multiple linear regression Adjusted B coefficient (S.E) | p-value | Simple linear regression Crude B coefficient (S.E) | p-value | Multiple linear regression Adjusted B coefficient (S.E) | p-value | Simple linear regression Crude B coefficient (S.E) | p-value | Multiple linear regression Adjusted B coefficient (S.E) | p-value | Simple linear regression Crude B coefficient (S.E) | p-value | Multiple linear regression Adjusted B coefficient (S.E) | p-value | Simple linear regression Crude B coefficient (S.E) | p-value | Multiple linear regression Adjusted B coefficient (S.E) | p-value |
| Knowledge of COVID-19 | - | - | - | - | 0.129 (0.029) | <0.001 | 0.113 (0.029) | <0.001 | 0052 (0.057) | 0.365 | - | - | -0.003 (0.033) | 0.930 | - | - | 0.026 (0.066) | 0.690 | - | - | 0.073 (0.039) | 0.063 | 0.070 (0.039) | 0.075 |
| Preventive measures | - | - | - | - | - | - | - | - | 0.022 (0.103) | 0.828 | - | - | 0.089 (0.060) | 0.140 | 0.059 (0.059) | 0.315 | - | - | - | - | - | - | - | - |
| Risk perception | - | - | - | - | - | - | - | - | - | - | - | - | - | - | - | - | 0.051 (0.063) | 0.415 | - | - | 0.043 (0.037) | 0.243 | 0.040 (0.037) | 0.286 |
| Preparedness and Self-efficacy | - | - | - | - | - | - | - | - | - | - | - | - | - | - | - | - | 0.182 (0.108) | 0.092 | 0.189 (0.109) | 0.083 | 0.028 (0.064) | 0.663 | - | - |
| Age | 0.109 (0.071) | 0.123 | 0.121 (0.070) | 0.084 | 0.011 (0.039) | 0.772 | - | - | 0.157 (0.074) | 0.035 | 0.130 (0.074) | 0.081 | 0.031 (0.043) | 0.479 | - | - | 0.081 (0.086) | 0.348 | - | - | -0.007 (0.051) | 0.896 | - | - |
| Female (Reference - males) | 0.620 (0.303) | 0.041 | 0.521 (0.303) | 0.086 | 0.522 (0.166) | 0.002 | 0.405 (0.165) | 0.014 | -0.667 (0.318) | 0.037 | -0.565 (0.313) | 0.072 | -0.204 (0.186) | 0.273 | - | - | -0.671 (0.369) | 0.070 | -0.509 (0.374) | 0.175 | 0.147 (0.219) | 0.503 | - | - |
| Married (Reference - Single and divorced) | 1.712 (1.605) | 0.287 | - | - | 1.019 (0.887) | 0.252 | - | - | 0.734 (1.689) | 0.664 | - | - | 2.763 (0.972) | 0.005 | 2.061 (1.215) | 0.091 | 2.209 (1.953) | 0.259 | - | - | 0.804 (1.158) | 0.488 | - | - |
| Level of education among students (Reference – Non Postgraduate) | -0.780 (0.991) | 0.432 | - | - | 0.607 (0.548) | 0.268 | - | - | 0.617 (1.042) | 0.554 | - | - | 1.141 (0.604) | 0.060 | 0.362 (0.747) | 0.628 | 1.133 (1.205) | 0.348 | - | - | -0.038 (0.715) | 0.958 | - | - |
| Medical faculty (Reference – Non-Medical Faculty) | 1.488 (0.488) | 0.002 | 1.239 (0.490) | 0.012 | 0.733 (0.271) | 0.007 | 0.453 (0.269) | 0.093 | -0.495 (0.519) | 0.341 | - | - | 0.219 (0.302) | 0.470 | - | - | -0.765 (0.601) | 0.204 | -0.742 (0.607) | 0.222 | 0.039 (0.357) | 0.914 | - | - |
| With chronic medical illness (Reference - no chronic medical illness) | 0.166 (1.249) | 0.894 | - | - | 0.754 (0.690) | 0.275 | - | - | 1.753 (0.1309) | 0.181 | 1.202 (1.283) | 0.349 | 0.953 (0.763) | 0.212 | 1.157 (0.748) | 0.123 | 2.290 (1.515) | 0.132 | 2.017 (1.512) | 0.183 | 1.282 (0.898) | 0.154 | 1.223 (0.896) | 0.174 |
| Tested and status positive (Reference - not tested, status unknown; tested, status negative) | - | - | - | - | - | - | - | - | - | - | - | - | - | - | - | - | - | - | - | - | - | - | - | - |
| Positive case(s) within social group (Reference - no positive cases) | 0.171 (0.331) | 0.606 | - | - | -0.143 (0.183) | 0.435 | - | - | 0.612 (0.346) | 0.078 | 0.632 (0.340) | 0.064 | -0.553 (0.200) | 0.006 | -0.527 (0.198) | 0.008 | 0.182 (0.402) | 0.652 | - | - | -0.073 (0.238) | 0.760 | - | - |
| Red zone (Reference - other than red zone) | 0.955 (0.331) | 0.004 | 0.900 (0.326) | 0.006 | -0.038 (0.185) | 0.838 | - | - | 0.334 (0.351) | 0.342 | - | - | -0.207 (0.205) | 0.312 | - | - | 0.126 (0.407) | 0.757 | - | - | 0.219 (0.241) | 0.365 | - | - |
| Stay alone (Reference – not staying alone) | 0.244 (0.992) | 0.806 | - | - | 0.479 (0.548) | 0.382 | - | - | -1.303 (1.040) | 0.211 | -1.225 (1.035) | 0.237 | 0.629 (0.606) | 0.300 | - | - | 1.005 (1.206) | 0.405 | - | - | 0.858 (0.714) | 0.230 | 0.912 (0.712) | 0.201 |
| Household with children (Reference - without children) | -0.150 (0.320) | 0.639 | - | - | 0.179 (0.177) | 0.313 | - | - | 0.285 (0.336) | 0.396 | - | - | 0.476 (0.194) | 0.015 | 0.432 (0.192) | 0.025 | -0.480 (0.388) | 0.217 | -0.552 (0.390) | 0.158 | -0.041 (0.230) | 0.858 | - | - |
| Household with elderly (Reference - without elderly) | -0.005 (0.322) | 0.988 | - | - | -0.089 (0.178) | 0.617 | - | - | 1.285 (0.331) | <0.001 | 1.146 (0.334) | 0.001 | -0.195 (0.197) | 0.323 | - | - | 0.112 (0.392) | 0.774 | - | - | -0.182 (0.232) | 0.433 | - | - |
| Model intercept | - | - | 15.948 (1.561) | <0.001 | - | - | 6.262 (0.549) | <0.001 | - | - | 7.247 (1.628) | <0.001 | - | - | 9.716 (0.522) | <0.001 | - | - | 5.734 (1.148) | <0.001 | - | - | 3.792 (0.810) | <0.001 |

| Supplementary Table S7: Factors associated with knowledge and preventive (own) measures towards COVID-19 in staffs (n = 93). | | | | | | | | | | | | | | | | | | | | | | | | |
|---|---|---|---|---|---|---|---|---|---|---|---|---|---|---|---|---|---|---|---|---|---|---|---|---|
| Variables | Knowledge of COVID-19 | | | | Preventive (own) measures | | | | COVID-19 risk perception (Aggregate score) | | | | Preparedness & perceived self-efficacy (Aggregate score) | | | | Unwanted behavior | | | | Desirable behavior | | | |
| | Simple linear regression Crude B coefficient (S.E.) | p-value | Multiple linear regression Adjusted B coefficient (S.E) | p-value | Simple linear regression Crude B coefficient (S.E) | p-value | Multiple linear regression Adjusted B coefficient (S.E) | p-value | Simple linear regression Crude B coefficient (S.E) | p-value | Multiple linear regression Adjusted B coefficient (S.E) | p-value | Simple linear regression Crude B coefficient (S.E) | p-value | Multiple linear regression Adjusted B coefficient (S.E) | p-value | Simple linear regression Crude B coefficient (S.E) | p-value | Multiple linear regression Adjusted B coefficient (S.E) | p-value | Simple linear regression Crude B coefficient (S.E) | p-value | Multiple linear regression Adjusted B coefficient (S.E) | p-value |
| Knowledge of COVID-19 | - | - | - | - | 0.109 (0.046) | 0.021 | 0.097 (0.044) | 0.030 | -0.018 (0.122) | 0.885 | - | - | 0.023 (0.075) | 0.761 | - | - | 0.134 (0.128) | 0.297 | - | - | 0.044 (0.078) | 0.578 | - | - |
| Preventive measures | - | - | - | - | - | - | - | - | -0.173 (0.268) | 0.519 | - | - | 0.322 (0.161) | 0.048 | 0.293 (0.166) | 0.080 | - | - | - | - | - | - | - | - |
| Risk perception | - | - | - | - | - | - | - | - | - | - | - | - | - | - | - | - | -0.082 (0.110) | 0.459 | - | - | -0.112 (0.066) | 0.093 | -0.045 (0.076) | 0.550 |
| Preparedness and Self-efficacy | - | - | - | - | - | - | - | - | - | - | - | - | - | - | - | - | 0.293 (0.177) | 0.101 | 0.246 (0.168) | 0.148 | 0.140 (0.109) | 0.200 | 0.082 (0.118) | 0.491 |
| Age | 0.035 (0.022) | 0.122 | 0.035 (0.022) | 0.122 | 0.001 (0.010) | 0.959 | - | - | -0.031 (0.026) | 0.232 | -0.007 (0.026) | 0.804 | 0.008 (0.016) | 0.610 | - | - | 0.033 (0.027) | 0.226 | 0.019 (0.026) | 0.472 | 0.008 (0.017) | 0.625 | - | - |
| Female (Reference - males) | 0.330 (0.578) | 0.569 | - | - | -0.111 (0.263) | 0.674 | - | - | 0.611 (0.672) | 0.366 | - | - | 0.105 (0.414) | 0.801 | - | - | -1.270 (0.697) | 0.072 | -0.841 (0.677) | 0.218 | -0.179 (0.432) | 0.679 | - | - |
| Married (Reference - Single and divorced) | 0.048 (0.542) | 0.930 | - | - | -0.116 (0.246) | 0.639 | - | - | -0.599 (0.629) | 0.344 | - | - | -0.043 (0.388) | 0.913 | - | - | 0.180 (0.664) | 0.786 | - | - | 0.731 (0.398) | 0.069 | 0.468 (0.431) | 0.281 |
| Highest education level obtained among administrative and academic staff (reference – Non postgraduate/professional) | -0.490 (0.543) | 0.369 | - | - | -0.527 (0.242) | 0.032 | -0.436 (0.237) | 0.069 | -0.474 (0.634) | 0.457 | - | - | -0.463 (0.387) | 0.234 | -0.309 (0.392) | 0.433 | 0.541 (0.666) | 0.419 | - | - | 0.398 (0.405) | 0.329 | - | - |
| Medical faculty (Reference – Non-Medical Faculty) | -0.336 (0.807) | 0.678 | - | - | -0.870 (0.356) | 0.016 | -0.731 (0.347) | 0.038 | 0.577 (0.940) | 0.541 | - | - | 0.340 (0.576) | 0.557 | - | - | -0.065 (0.989) | 0.948 | - | - | -0.741 (0.598) | 0.219 | -0.953 (0.584) | 0.107 |
| With chronic medical illness (Reference - no chronic medical illness) | 1.261 (1.261) | 0.345 | - | - | 0.601 (0.604) | 0.322 | - | - | -3.132 (1.521) | 0.042 | -2.693 (1.537) | 0.083 | 0.831 (0.950) | 0.384 | - | - | 0.551 (1.634) | 0.737 | - | - | 0.022 (0.997) | 0.982 | - | - |
| Tested and status positive (Reference - not tested, status unknown; tested, status negative) | 0.967 (2.622) | 0.713 | - | - | 1.087 (1.189) | 0.363 | - | - | 3.793 (3.035) | 0.215 | 3.181 (2.916) | 0.278 | 1.815 (1.867) | 0.334 | - | - | 10.641 (3.017) | 0.001 | 8.911 (3.099) | 0.005 | 3.054 (1.934) | 0.118 | 3.256 (1.935) | 0.096 |
| Positive case(s) within social group (Reference - no positive cases) | 0.129 (0.647) | 0.842) | - | - | -0.272 (0.293) | 0.356 | - | - | 0.972 (0.748) | 0.197 | 0.743 (0.721) | 0.306 | -0.079 (0.463) | 0.864 | - | - | -0.427 (0.792) | 0.592 | - | - | 0.274 (0.483) | 0.572 | - | - |
| Red zone (Reference - other than red zone) | 0.054 (0.671) | 0.936 | - | - | -0.491 (0.301) | 0.106 | -0.592 (0.284) | 0.040 | -0.483 (0.781) | 0.538 | - | - | 0.124 (0.480) | 0.796 | - | - | -1.390 (0.810) | 0.089 | -0.705 (0.797) | 0.379 | -0.622 (0.497) | 0.214 | -0.436 (0.489) | 0.376 |
| Stay alone (Reference – not staying alone) | 1.023 (1.096) | 0.353 | - | - | -0.454 (0.499) | 0.366 | - | - | -0.264 (1.285) | 0.837 | - | - | 0.672 (0.785) | 0.394 | - | - | 0.207 (1.350) | 0.879 | - | - | -0.690 (0.820) | 0.402 | - | - |
| Household with children (Reference - without children) | -0.119 (0.546) | 0.828 | - | - | -0.263 (0.247) | 0.291 | - | - | -1.794 (0.609) | 0.004 | -1.641 (0.614) | 0.009 | 0.300 (0.390) | 0.444 | - | - | 0.398 (0.669) | 0.553 | - | - | 1.178 (0.389) | 0.003 | 0.986 (0.434) | 0.026 |
| Household with elderly (Reference - without elderly) | -0.355 (0.573) | 0.537 | - | - | -0.129 (0.261) | 0.622 | - | - | 0.694 (0.666) | 0.300 | - | - | 0.032 (0.411) | 0.938 | - | - | 0.597 (0.701) | 0.397 | - | - | -0.210 (0.428) | 0.626 | - | - |
| Model intercept | - | - | 17.779 (0.852) | <0.001 | - | - | 7.881 (0.906) | <0.001 | - | - | 11.105 (1.006) | <0.001 | - | - | 7.758 (1.554) | <0.001 | - | - | 5.308 (2.091) | 0.013 | - | - | 5.400 (1.804) | 0.004 |

| Supplementary Table S8: Summary of results on knowledge, risk perception, preparedness & perceived self-efficacy, preventive measures, and behavior related to COVID-19 between genders (n = 434) . | | | | | |
|---|---|---|---|---|---|
| **Assessment (minimum and maximum score)** | **Category** | **All (n = 434)** | **Male (n=172)** | **Female (n = 262)** | **p-values** |
| **Knowledge on COVID-19** (0-22) | Mean ± SD | 18.72±2.73 | 18.48±2.79 | 18.91±2.69 | 0.105 |
| **COVID-19 risk perception** | | | | | |
| Aggregate score<br>(3-21) | Mean ± SD | 10.27±2.93 | 10.51±2.96 | 10.10±2.91 | 0.150 |
| Probability of infection<br>(1=extremely unlikely; 7=extremely likely) | Mean ± SD | 3.38±1.37 | 3.47±1.50 | 3.32±1.28 | 0.299 |
| Susceptibility to the disease<br>(1=not at all susceptible; 7=very susceptible) | Mean ± SD | 3.49±1.37 | 3.54±1.47 | 3.45±1.31 | 0.522 |
| Severity of illness<br>(1=very strongly disagree; 7=very strongly agree) | Mean ± SD | 3.40±1.49 | 4.49±1.56 | 4.67±1.43 | 0.210 |
| **Preparedness & perceived self-efficacy** | | | | | |
| Aggregate score<br>(2-14) | Mean ± SD | 10.25±1.73 | 10.34±1.82 | 10.19±1.67 | 0.389 |
| Protection ability<br>(1=not at all;<br>7=very much so) | Mean ± SD | 5.68±0.99 | 5.69±1.04 | 5.67±0.96 | 0.806 |
| Avoidance ability<br>(1=extremely difficult; 7=extremely easy) | Mean ± SD | 4.57±1.20 | 4.65±1.29 | 4.52±1.14 | 0.299 |
| **Preventive (own) measures**<br>(0-10) | Mean ± SD | 8.71±1.47 | 8.47±1.64 | 8.88±1.32 | **0.004** |
| **Unwanted behaviours**<br>(0-18) | Mean ± SD | 7.21±3.33 | 7.67±3.51 | 6.91±3.17 | **0.019** |
| **Desirable behaviours**<br>(0-9) | Mean ± SS | 5.63±1.99 | 5.56±2.00 | 5.68±1.98 | 0.568 |
| Data are presented either in mean ± standard deviation (SD), median (interquartile range), range, frequency (%). | | | | | |
